# Spotted Fever Group *Rickettsia* sp. Closely Related to *R. japonica,* Thailand

**DOI:** 10.3201/eid1504.081271

**Published:** 2009-04

**Authors:** Nobuhiro Takada, Hiromi Fujita, Hiroki Kawabata, Shuji Ando, Akiko Sakata, Ai Takano, Udom Chaithong

**Affiliations:** University of Fukui, Fukui, Japan (N. Takada); Ohara General Hospital, Fukushima, Japan (H. Fujita); National Institute of Infectious Diseases, Tokyo, Japan (H. Kawabata, S. Ando, A. Sakata, A. Takano); Faculty of Medicine, Chiang Mai University, Chiang Mai, Thailand (U. Chaithong)

**Keywords:** Rickettsia, spotted fever, Rickettsia japonica, Thailand, letter

**To the Editor**: In response to a recent report that suggested human infection with *Rickettsia japonica* in northeastern Thailand (*1*), we phylogenetically reexamined spotted fever group rickettsiae (SFGR) from Thailand. The organism had been isolated from a male *Haemaphysalis hystricis* tick found on Mt. Doi Suthep, Chiang Mai, northern Thailand, in December 2001. The strain was designated TCM1 and was not distinguishable from *R. japonica* by indirect immunoperoxidase stain using monoclonal antibody (*2*).

After propagating strain TCM1 in L-929 cell culture, we extracted DNA by using a Wizard Genomic DNA Purification Kit (Promega, Madison, WI, USA). We subjected the DNA to sequencing that targeted a 491-bp fragment of rickettsial outer membrane protein A (*ompA*), a 394-bp fragment of the rickettsial genus–specific 17-kDa antigen gene, and a 1,250-bp fragment of citrate synthase gene (*gltA*). Direct sequencing of amplicons was performed as previously described (*3*). Phylogenetic analyses based on o*mpA* indicated that strain TCM1 was closely related to and clustered within the same clade as *R. japonica* strain YH (98.4% identity) (Figure, panel A). Also, a 17-kDa antigen gene obtained from strain TCM1 showed 99.5% identity to the corresponding gene of *R. japonica* (Figure, panel B). Our phylogenetic analysis with *ompA* and 17-kDa antigen gene showed that strain TCM1 was closely related to *R. japonica* but distinguished from *Rickettsia* sp. PMK94 (which was closely related to *R. heilongjiangensis* from northeastern China) (*3*); another SFGR agent, *R. honei* from *Ixodes granulatus* ticks in Thailand (*4*), was apparently different from strain TCM1 (Figure). Phylogenetic analyses based on *gltA* (99.4%–99.6% identity) showed that strain TCM1 is also closely related to *R. japonica* and *Rickettsia* sp. strain PMK94 (data not shown). Thus, we describe the *R. japonica* group in Thailand. DNA sequences of strain TCM1 were determined and deposited in GenBank/EMBL/DDBJ under the following accession nos.: *ompA,* AB359459; 17-kDa antigen, AB359457; *gltA,* AB359458.

**Figure:**
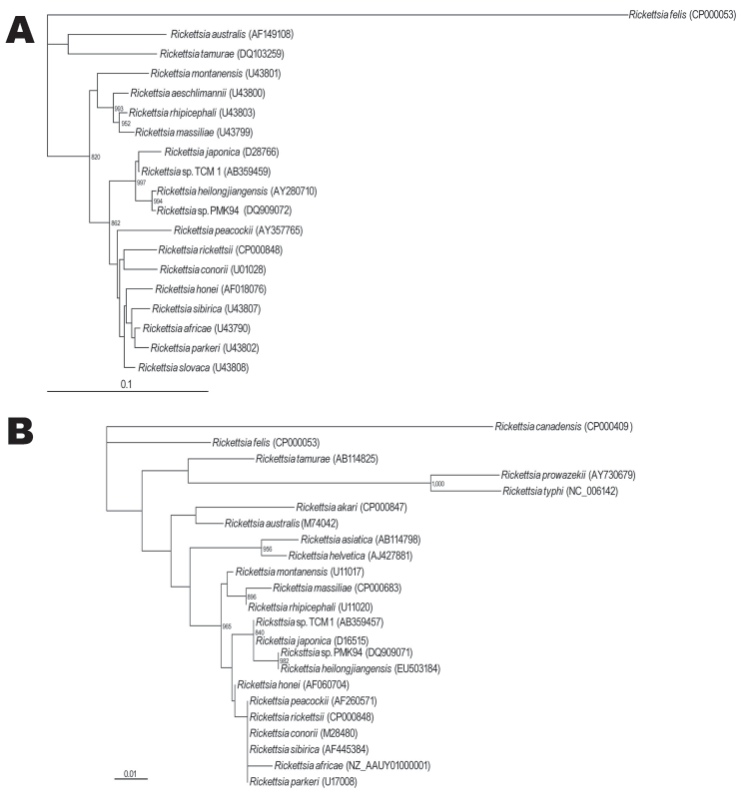
Phylogenetic analysis based on *ompA* gene (A) and rickettsial genus–specific 17-kDa antigen gene (B). Sequences were aligned by using the ClustalW software package (http://clustalw.ddbj.nig.ac.jp/top-j.html), and neighbor-joining phylogenetic tree construction and bootstrap analysis were conducted according to the Kimura 2-parameter method (www.ddbj.nig.ac.jp). Pairwise alignments were performed with an open-gap penalty of 10, a gap extension penalty of 0.5, and a gap distance of 8. Multiple alignments were also performed with the same values, and the phylogenetic branches were supported by bootstrap analysis with 1,000 replications (>800 were indicated). *Rickettsia felis* (CP000053) and *R. canadensis* (CP000409) were used as outgroups for *ompA* and 17-kDa antigen gene, respectively. The phylogenetic tree was constructed by using TreeView software version 1.5 (http://taxonomy.zoology.gla.ac.uk/rod/treeview.html). Scale bars indicate nucleotide substitutions (%) per site.

*R. japonica* is the specific pathogen of Japanese spotted fever, which has been found mainly in southwestern Japan (*5*). The present strain, closely related to *R. japonica,* is likely to have been isolated from *H. hystricis* in Thailand because *R. japonica* frequently has been isolated, or detected by PCR, from the same tick species in Japan (*6*). Such tick species–specificity of SFGR should be considered when speculating on any geopathologic relationships of rickettsioses among different SFGR-endemic areas. Previous reports on spotted fever–positive results of human serosurveys (*7*,*8*) and on a clinical case (*9*) in northern Thailand may provide epidemiologic background. In Asia, multiple species of rickettsiea (e.g., *R. japonica, R. heilongjiangensis, R. honei*) are the causative agents of spotted fever rickettsioses, so the agent closely related to *R. japonica* could cause spotted fever in Thailand. Additionally, *R. japonica* has been found in Korea (*10*), and our current study indicates that *R. japonica* and its genetic variants are widely distributed in Far Eastern countries, including Japan (Grant-in-Aid for International Cooperative Research, unpub. data). Therefore, the epidemiology and genetic variation of SFGR throughout Asia should be examined by molecular studies.
